# Fulminant Invasive Aspergillosis in a Previously Healthy Woman After Cesarean Section and a Review of the Literature

**DOI:** 10.3390/idr16060100

**Published:** 2024-12-20

**Authors:** Luca Pipitò, Gennaro Baldino, Giovanni Bartoloni, Maurizio Sanguinetti, Elvira Ventura Spagnolo, Antonio Cascio

**Affiliations:** 1 Department of Health Promotion, Mother and Child Care, Internal Medicine and Medical Specialties G D’Alessandro, University of Palermo, 90133 Palermo, Italy; 2 Infectious and Tropical Diseases Unit, Sicilian Regional Reference Center for the Fight Against AIDS, AOU Policlinico P. Giaccone, 90127 Palermo, Italy; 3 Legal Medicine Section, Biomorf Department, Messina University, 98122 Messina, Italy; dott.gennarobaldino@gmail.com (G.B.); venturaspagnoloelvira@gmail.com (E.V.S.); 4 Department of Pathology, Garibaldi Hospital ARNAS Garibaldi, 95125 Catania, Italy; 5 Department of Laboratory and Infectious Sciences, Fondazione Policlinico Universitario A. Gemelli IRCCS, L.go A. Gemelli 8, 00168 Rome, Italy; maurizio.sanguinetti@unicatt.it

**Keywords:** pregnancy, invasive pulmonary aspergillosis, brain aspergillosis, myocardial aspergillosis

## Abstract

**Background:** Invasive aspergillosis is an extremely rare condition in healthy and immunocompetent individuals, and very few cases have been reported in previously healthy, pregnant, or postpartum women. **Method:** We describe a case of invasive aspergillosis in a puerperal patient and present literature review results. **Case:** We present a case of fulminant invasive pulmonary aspergillosis with cerebral, cardiac, and gastric involvement in a young woman, occurring a few days after an elective cesarean section. The patient succumbed after intensive care unit admission, and the diagnosis was made postmortem through autopsy. **Conclusion:** A total of 20 cases of invasive aspergillosis have been previously reported in pregnant or postpartum women, with high mortality. The risk of opportunistic fungal infections during pregnancy and postpartum should not be underestimated.

## 1. Introduction

Aspergillosis refers to a spectrum of conditions caused by fungi belonging to the *Aspergillus* species, encompassing both non-invasive and invasive diseases [1]. Non-invasive forms include allergic bronchopulmonary aspergillosis and allergic fungal rhinosinusitis, while invasive forms include chronic pulmonary aspergillosis and invasive pulmonary aspergillosis (IPA) [1]. *Aspergillus* spp. is a filamentous fungus ubiquitous in the environment, found both indoors and outdoors—in soil, dust, water, decaying vegetation, fertilizers, certain foods and spices, and ventilation ducts. The fungus disseminates spores (conidia with a diameter of 2–5 μm, which humans commonly inhale into the lower respiratory tract) into the air [2]. Under normal conditions, conidia are phagocytosed by polymorphonuclear cells and macrophages, preventing disease development in healthy, immunocompetent individuals [2]. Natural killer cells control fungal proliferation in the lungs through both direct and indirect mechanisms of killing invading organisms. Adaptive immune cells, including Th1 and Th17 cells, contribute to antifungal defense by producing their signature cytokines, interferon-γ and interleukin-17, respectively [3]. Additionally, lung epithelial cells play a role in resistance to fungal infections through the internalization of pathogens, production of inflammatory cytokines, and secretion of antimicrobial peptides [3]. In immunocompromised individuals, such as neutropenic patients, transplant recipients, or hematological patients, defects in cell-mediated immunity promote the development of invasive forms, which are associated with a poor prognosis [1,2]. The primary risk factors for IPA include severe or prolonged neutropenia and defects in cell-mediated immunity [1,2]. However, rare cases of IPA in immunocompetent individuals have been documented, especially under certain predisposing conditions. Factors such as severe influenza [4,5], chronic lung diseases [6], surgical site infection [7], and sepsis [8] can predispose otherwise healthy individuals to IA. Although the likelihood of invasive disease remains significantly lower in immunocompetent patients, these cases highlight the importance of understanding host immune responses and identifying underlying vulnerabilities that may compromise resistance to *Aspergillus* infections. More recently, immunocompetent patients with severe SARS-CoV2 have been reported to be affected by COVID-19-associated pulmonary aspergillosis in the absence of conventional risk factors for invasive aspergillosis [9]. Furthermore, a score for predicting IPA in immunocompetent critically ill patients has been developed, including a higher C-reactive protein–albumin ratio, chronic obstructive pulmonary disease, continuous renal replacement therapy, a high dose of corticosteroids, broad-spectrum antibiotics, and blood galactomannan positivity as risk factors [10]. In any case, invasive aspergillosis remains extremely rare in subjects without severe immune deficits and even more so in fulminant cases with multi-organ involvement [11,12]. Here, we report a rare case of fulminant and fatal IPA with myocardial and cerebral involvement that developed in a previously healthy woman following a cesarean section, and review the literature.

## 2. Case Description

### 2.1. Presentation

A previously healthy 40-year-old woman was admitted to the emergency department in April 2024 with complaints of abdominal pain, diarrhea, and malaise. Her medical history included a cesarean section performed four days earlier, insulin-dependent diabetes mellitus, class 1 obesity (body mass index of 34.3 kg/m^2^), and post-traumatic mixed anxiety–depressive disorder, which developed after experiencing four miscarriages between 2016 and 2019. The cesarean section was scheduled based on psychiatric recommendations due to the patient’s obstetric history and anxiety–depressive disorder. No complications were reported during the cesarean, and the patient was discharged postoperatively without incident. On physical examination, the patient presented with abdominal pain and tenderness, along with signs of dehydration, including dry skin and mucous membranes. Vital signs revealed a heart rate (HR) of 140 beats per minute (bpm), blood pressure (BP) of 119/74 mmHg, and oxygen saturation of 97% on room air. She was hypothermic, with a body temperature of 35 °C.

### 2.2. Investigations

Laboratory tests revealed acute, profound leukopenia and elevated C-reactive protein (CRP) and procalcitonin (PCT) levels (Table 1).

The previous day’s blood work showed a normal white blood cell count (8960 cells/μL, with 77.6% neutrophils). Testing for antibodies against HIV was negative. The patient’s condition rapidly worsened, with a subsequent drop in blood pressure to 85/57 mmHg. She was transferred to the intensive care unit (ICU) with clinical suspicion of septic shock and impending multiorgan failure (MOF). Blood and urine cultures were obtained, and empiric broad-spectrum antibiotic therapy was initiated with piperacillin/tazobactam and ciprofloxacin.

Computed tomography (CT) of the abdomen revealed an enlarged uterus with thickened, heterogeneous walls consistent with recent post-cesarean changes. Additionally, small gas bubbles were noted within the uterine tissue, along with significant gastric distension with fluid levels, ascites in the abdominopelvic region, gaseous distension of the transverse colon, and hydro-gaseous distension of small bowel loops, particularly in the lower abdomen. An exploratory laparotomy performed the same day revealed peritonitis, with ecchymotic suffusion of the viscera, parietal, and visceral peritoneum, and retraction and cyanosis of the omentum. Microbiological cultures from blood, urine, and peritoneal seropurulent fluid were negative. A brain CT scan showed no abnormalities, while a chest CT scan revealed moderate bilateral pleural effusions, extensive atelectasis, areas of consolidation in the right upper lung lobe, and confluent ground-glass opacities in the apical-dorsal segment of the left upper lobe. However, no findings suggestive of pneumonia, aside from pleural effusion, were observed on a follow-up CT scan performed seven days later. The patient was connected to a ventilatory prosthesis in SIMV-PC mode (16 breaths per 7 min). Monitoring showed HR 130 bpm; BP 120/75 mmHg; SpO_2_ 92%. Five days after admission, the patient developed disseminated intravascular coagulation (DIC) with severe thrombocytopenia (a platelet count as low as 3000/μL) and subsequent necrosis of the toes on her right foot. She required hemodialysis and multiple transfusions of fresh frozen plasma. Transthoracic echocardiography ruled out endocarditis. During the hospital stay, the patient continued mechanical ventilation and analgesic sedation, maintained a Glasgow Coma Scale score of 4 points (1 + 2 + 1), and exhibited hemodynamic instability under amine therapy, characterized by hypotension and tachycardia.

**Figure 1:**
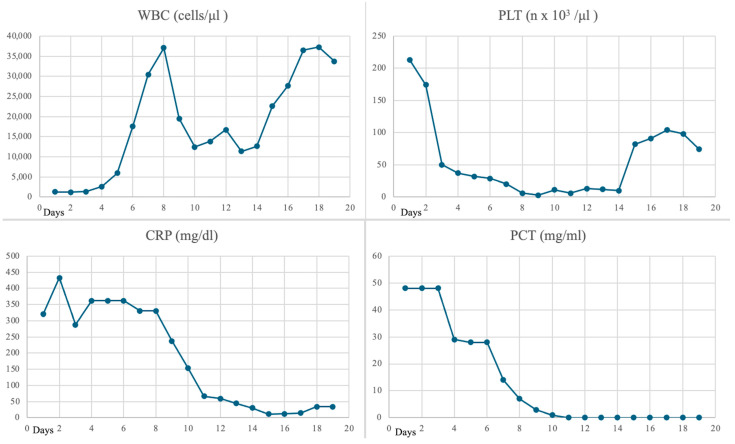
Trends of white blood cell (WBC), platelet (PLT), C-reactive protein (CRP), and procalcitonin (PCT) values during ICU hospitalization.

Despite broad-spectrum antibiotics, vasopressor support with noradrenaline and dopamine, and improvement in laboratory parameters (including a rapid increase in white blood cell count and a significant reduction in inflammatory markers, Figure 1), the patient’s clinical condition deteriorated, and she experienced intermittent fever and hypothermia. Corticosteroid therapy was administered for the entire duration of the hospital stay (methylprednisolone, 40 mg twice daily for 19 days).

**Figure 2:**
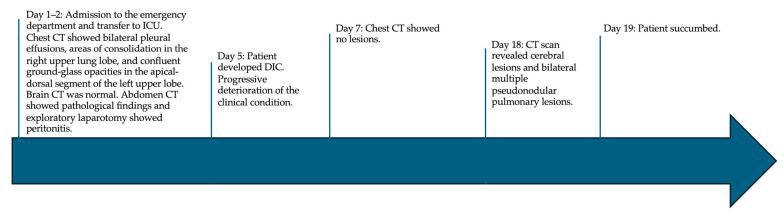
Timeline of patient’s clinical history. CT: computed tomography.

A follow-up CT scan, performed 18 days after admission, revealed multiple new confluent hypodense areas in the cerebellar and cerebral hemispheres and bilateral multiple pseudonodular pulmonary lesions, some cavitated and partly confluent with air bronchograms. The patient succumbed the following day. A timeline of the patient’s history is depicted in Figure 2. 

### 2.3. Autopsy

**Figure 3:**
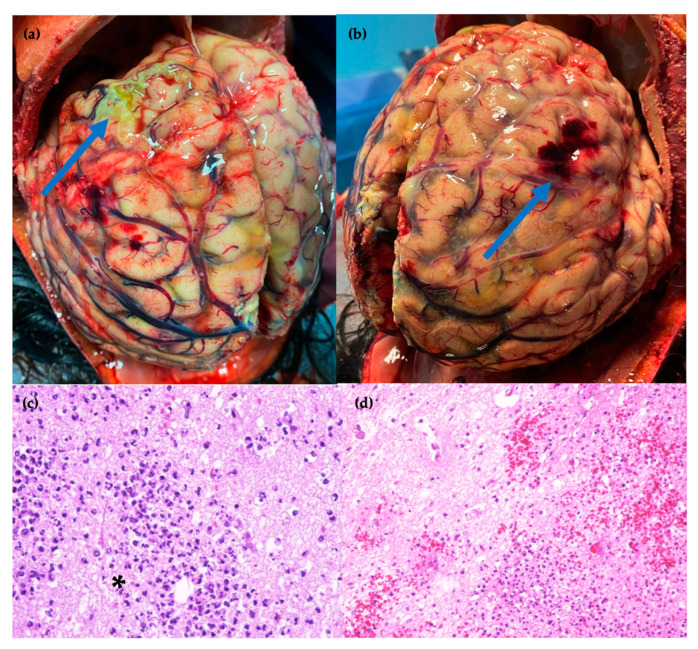
Brain sites of (**a**) widespread areas of yellowish purulent exudates filling subarachnoid spaces; (**b**) reddish temporal subarachnoid hemorrhagic infarct, typical of fungal infection (arrow); (**c**) hematoxylin and eosin (HE) stain × 40 magnification: mycotic abscess with hyphal fragments (*); and (**d**) HE stain × 20 magnification: numerous petechial necrotic hemorrhagic foci.

**Figure 4:**
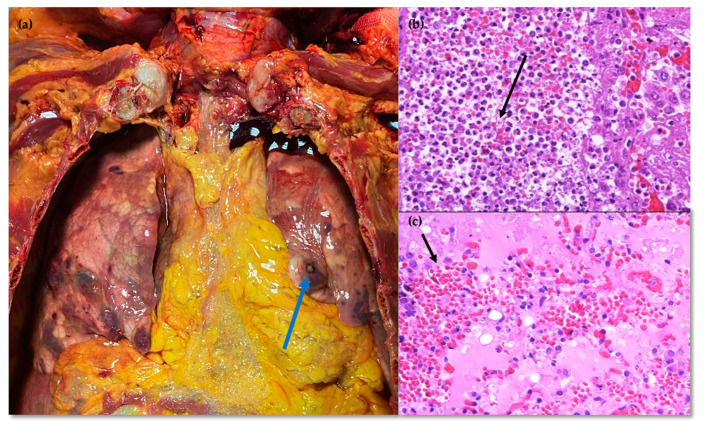
Lung (**a**) site of gross, yellowish, subserosal micro-nodular abscess; (**b**) histological section HE stain × 40 magnification: abscess centered by fungal hyphal tangles (arrow), ascribable to *Aspergillus* spp.; and (**c**) histological section HE stain × 40 magnification: massive hemorrhagic pulmonary edema (arrow).

**Figure 5:**
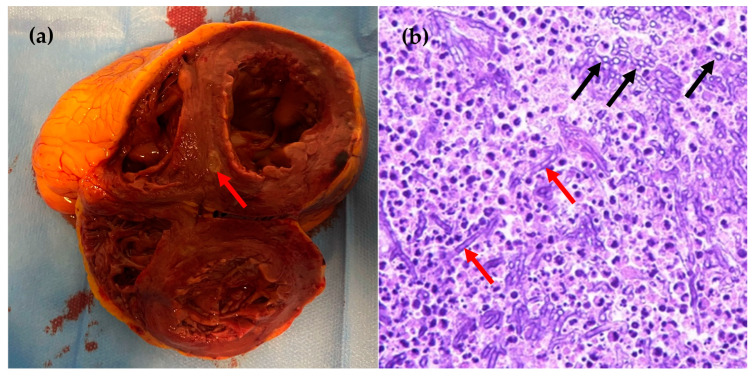
Heart (**a**) abscess, 0.5 cm in diameter, in transverse slice of intraventricular septum; and (**b**) histological section HE stain × 40 magnification: necrotizing mycotic microabscess consisting of septate hyphae (red arrows) and spores (black arrows).

An autopsy was performed, revealing significant findings in the brain (Figure 3), lungs (Figure 4), and heart (Figure 5). In the brain, there was septic mycotic subocclusion of intraparenchymal arterioles up to 2 mm in diameter, cerebral microabscesses in the early exudative phase, and diffuse petechial necro-hemorrhagic foci. The lungs showed septic fungal necrotizing vasculitis, multiple abscesses centered around fungal hyphae, multifocal septic thromboembolism, and septic fungal lymphadenitis. In the heart, numerous necrotizing mycotic microabscesses composed of hyphae and spores were found between myocardial fibers, with multiple necro-hemorrhagic foci in all other organs. Abundant infiltrating hyphae were highlighted in the stomach submucosa. DNA extraction from paraffin-embedded tissue samples and real-time PCR for *Aspergillus* spp. were performed on brain, lung, and heart specimens, yielding positive results with 298 copies/mL, 643 copies/mL, and 4154 copies/mL, respectively.

## 3. Materials and Methods

In addition to the case described in this article, we conducted a literature search on invasive aspergillosis associated with pregnancy using only the PubMed database. We employed the search strings “(Aspergillosis OR *Aspergillus*) AND (pregnancy OR pregnant OR postpartum OR puerperal OR puerperium) AND (case report OR case series OR clinical cases)”. No language and temporal restrictions were applied during the research. We yielded 17 relevant articles.

## 4. Discussion

Invasive aspergillosis (IA) is rarely reported in immunocompetent patients. Pregnancy is generally considered an immunomodulated state with increased susceptibility to infections due to alterations in the immune response, notably a reduction in cellular immunity and a relative increase in humoral immunity [13].

The maternal immune system undergoes significant changes during pregnancy to tolerate the semi-allogeneic fetus. This includes a shift toward a Th2-dominant immune response, reduced Th1 activity, and alterations in innate immunity, such as impaired neutrophil and natural killer cell function [14]. Additionally, hormonal changes, particularly increased progesterone and cortisol levels, can modulate immune responses [14,15]. These adaptations are critical for maintaining fetal tolerance, but may compromise the host’s ability to combat certain pathogens, including fungi. As a result, pregnant women may be more susceptible to opportunistic infections such as IA. However, IA during pregnancy in previously healthy women remains extremely uncommon, with fulminant cases, such as ours, being exceptional.

We conducted a comprehensive review of the literature on cases of IA associated with pregnancy, identifying 15 case reports [16,17,18,19,20,21,22,23,24,25,26,27,28,29,30] and two case series [31,32]. Both case series detailed patients from a specific cluster in Sri Lanka, though the study by Rodrigo et al. included one additional patient [32]. Additionally, Lokuhetty et al. documented an autopsy of one of the patients from this case series [26]. Our review encompasses 20 patients, 8 during pregnancy and 12 postpartum. The results are summarized in Table 2.

### 4.1. Invasive Aspergillosis During Pregnancy

Invasive aspergillosis was documented in eight pregnant patients [18,19,21,22,23,24,28,30], resulting in two maternal deaths [18,22] and two fetal deaths [22,24]. One case lacked maternal outcome data [24], and one case resulted in a spontaneous abortion [21]. Several underlying risk factors or complications during pregnancy were noted, including eclampsia [18], tuberculosis [18,19], systemic lupus erythematosus [19], a prior history of sinus surgery and untreated left upper eyelid swelling [21], agranulocytosis related to autoimmune aplastic anemia [28], and chronic granulomatous disease (autosomal recessive NCF1 [p47phox]–deficient) diagnosed at age nine [30]. In three cases, no predisposing conditions or complications during pregnancy were reported [22,23,24].

The timing of IA onset varied: three cases were reported in the second trimester [21,22,28], three in the third trimester [18,19,23], one in the first trimester [30], and one where the trimester was not specified [24]. The infection site differed among cases, with five cases affecting the lungs [19,22,23,28,30], four involving the brain [18,21,22,23], one with fibrosing mediastinitis [18], one case of placental involvement [24], one instance of invasive sinusitis with intracranial extension [21], one maxillary sinusitis with periorbital cellulitis [28], and one liver involvement [22].

Early diagnosis and treatment initiation were recorded in two cases, including the use of voriconazole in one case [21] and liposomal amphotericin B followed by oral voriconazole in another [28]. Both patients survived: in one case, a spontaneous abortion occurred [21], while the other patient delivered a healthy infant, with no neurological or developmental issues observed at the six-month follow-up (the first reported use of voriconazole during pregnancy) [28]. *Aspergillus species* were identified in four cases: *Aspergillus fumigatus* in two cases [23,30], *Aspergillus flavus* in one case [28], and *Aspergillus niger* in another [24]. A case not included in our review reported a non-invasive aspergillosis characterized by a left eye lower eyelid pyogenic granuloma [33]

### 4.2. Aspergillus Meningitis Secondary to Spinal Anesthesia

Six cases of IA were documented in the postpartum period, linked to spinal anesthesia administered during cesarean sections as part of an outbreak in Sri Lanka in 2005. These cases were attributed to accidental inoculation of *Aspergillus* into the subarachnoid space, leading to meningitis. Three of these patients succumbed to the infection, with *Aspergillus fumigatus* confirmed through post-mortem cultures in each case [26,31,32].

### 4.3. Postpartum Invasive Aspergillosis

The remaining six postpartum cases occurred in patients with various underlying conditions. Documented underlying factors included drug-induced neutropenia [16], HELLP syndrome [17], tuberculosis [20,25], preeclampsia and aspiration pneumonia [25], and puerperal sepsis [27]. Surgical interventions were reported in three cases: one involving a cesarean section [25], another a hysterectomy for uterine hemorrhage [17], and a third involving a partial hysterectomy [27].

Regarding organ involvement, the lungs were affected in five cases [16,17,20,25,27]. One case presented as a disseminated infection involving the brain, heart, stomach, esophagus, trachea, and thyroid [17]. Mycotic aneurysm of the aortic arch and tracheobronchitis were reported in a single case each [25,29]. Four patients died [16,17,20,25], and a diagnosis was made post-mortem in one case [17]. *Aspergillus fumigatus* was identified in six cases [16,20,25,26,27,29].

### 4.4. Our Case

In our case, immunosuppression secondary to sepsis following a cesarean section, along with prolonged corticosteroid use, likely facilitated the development of invasive aspergillosis, although the rapid progression of the disease remains atypical and poorly understood. The patient developed MOF and DIC with severe tissue necrosis. Blood cultures were negative for bacterial pathogens, and it remains unclear whether aspergillosis was present subclinically before her ICU admission or if it emerged acutely during her stay. Despite a reduction in CRP and PCT levels with antibiotic therapy, her condition did not improve, and she succumbed within three weeks. IA with brain, lung, heart, and stomach involvement was confirmed during the autopsy. Moreover, acute IA of the heart is exceptionally rare [34], and only one previous case was reported in a puerperal woman [17]. Both insulin-dependent diabetes mellitus and class 1 obesity are well-documented to impair immune function [12,35,36]. Diabetes mellitus, especially when poorly controlled, can lead to hyperglycemia, which negatively affects neutrophil function, reducing chemotaxis, phagocytosis, and microbial killing [12,35]. Obesity, on the other hand, is associated with chronic low-grade inflammation and altered immune responses, including impaired macrophage and T-cell function, which may further compromise antifungal immunity [36]. In combination, these comorbidities may have acted synergistically to weaken the patient’s immune system, rendering them more vulnerable to IPA. The post-C-section status adds another layer of complexity, as surgical stress and recovery could exacerbate immune dysfunction.

We cannot rule out potential genetic susceptibility factors for IA, such as polymorphisms in genes encoding pentraxin-3, toll-like receptors 2 and 4, and dectin-1 [1]. However, very limited data are available in immunocompetent patients due to the rarity of IA in this population. The type and extent of immunosuppression appear to play a critical role in the development of invasive aspergillosis, sometimes in combination with predisposing genetic factors [37]. A previous study reported that TNFR1 influences IPA risk in hematological patients [38]. Additionally, increased susceptibility to aspergillosis has been observed in hematopoietic stem cell transplant patients with gene variants in TLR4 [39], plasminogen [40], PFKFB3 [41] polymorphisms, and other genetic factors [42].

## 5. Conclusions

In conclusion, the risk of opportunistic fungal infections during pregnancy and postpartum should not be underestimated. Although invasive aspergillosis is a very rare condition in immunocompetent individuals, pregnancy may, through mechanisms that are not yet fully understood, predispose individuals to this type of infection. If not diagnosed promptly, invasive aspergillosis during pregnancy can lead to unfavorable outcomes. Aspergillosis should be considered in cases of unexplained decline in general health when other etiologies have been ruled out, as well as in the differential diagnosis of new pulmonary or cerebral lesions.

## Figures and Tables

**Table 1:** Patient’s examination upon admission to emergency department.

Laboratory Analysis	Patient’s Result	Reference Range
WBC (cells/μL)	1290	4000–10,000
Neutrophils (%)	85	50–70
Lymphocytes (%)	12.4	20–40
Monocytes (%)	1.2	3–12
PLT (n/μL)	213,000	120,000–450,000
Hematocrit (%)	41.5	36–54
Hb (g/dL)	14.2	11.5–16
ALT (U/L)	23	0–35
AST (U/L)	9	0–35
GGT (U/L)	3	12–64
Bilirubin (mg/dL)	0.56	<1.2
Creatinine (mg/dL)	2.9	0.57–1.11
LDH (mg/dL)	428	125–220
CRP (mg/L)	320	<5
PCT (mg/mL)	48.24	<0.5
Fibrinogen (mg/dL)	715	140–450

WBC: white blood count, PLT: platelets, ALT: alanine transaminase, AST: aspartate aminotransferase, GGT: gamma-glutamyl transferase, LDH: lactate dehydrogenase, CRP: C-reactive protein, PCT: procalcitonin.

**Table 2:** Summary of previous case reports of invasive aspergillosis associated with pregnancy.

Authors	Gestational Period	Risk Factors or Complication	Organ Involvement	*Aspergillus* Species	Outcome
Aoki et al. [16]	Postpartum	Drug-induced neutropenia	Lungs	*A. fumigatus*	Dead
Kobayashi et al. [17]	Postpartum	HELLP syndrome, hysterectomy for uterine hemorrhage	Lungs, brain, heart, stomach, esophagus, trachea, thyroid	Not available	Dead
Vaideeswar et al. [18]	Third trimester	Eclampsia, Tuberculosis	Brain, fibrosing mediastinitis	Not available	Dead
Abd Rahman et al. [19]	Third trimester	Tuberculosis, systemic lupus erythematosus	Lungs	Not available	Maternal recovery and preterm birth
Ray et al. [20]	Postpartum	Tuberculosis	Lungs	*A. fumigatus*	Dead
Alsulaiman et al. [21]	Second trimester	Prior history of surgery	Brain, invasive sinusitis with intracranial extension	Not available	Maternal recovery and spontaneous abortion
Klock et al. [22]	Second trimester	None	Lungs, brain, liver	Not available	Maternal and fetal death
Pagliano et al. [23]	Third trimester	None	Lungs, brain	*A. fumigatus*	Maternal recovery
Ben Rejeb et al. [24]	Trimester was not specified	None	Placenta	*A. niger*	Fetal death
Stemmet et al. [25]	Postpartum	Tuberculosis, preeclampsia, aspiration pneumonia, cesarean section	Lungs, mycotic aneurysm of the aortic arch	*A. fumigatus*	Dead
Lokuhetty et al. [26]	Postpartum	Inoculation during spinal anesthesia	Brain	*A. fumigatus*	Dead
Briones-Claudett et al. [27]	Postpartum	Puerperal sepsis, partial hysterectomy	Lungs	*A. fumigatus*	Maternal recovery
Shoai et al. [28]	Second trimester	Aplastic anemia	Lungs, maxillary sinusitis with periorbital cellulitis	*A. flavus*	Maternal recovery
Shah et al. [29]	Postpartum	None	Pseudomembranous aspergillus tracheobronchitis	*A. fumigatus*	Lost to follow-up
Johnson et al. [30]	First trimester	Chronic granulomatous disease	Lungs	*A. fumigatus*	Maternal recovery

## Data Availability

There is no further data available.
